# A liposomal formulation of cyclosporine a shows promising results in treating symptoms of moderate to severe dry eye disease in dogs

**DOI:** 10.1007/s13346-025-02014-4

**Published:** 2026-01-30

**Authors:** María Ángela Caballo-González, Miguel Gómez-Ballesteros, Marco Brugnera, José Manuel Benítez-del-Castillo, Elisa Margarita González-Alonso-Alegre, Alfonso Rodríguez-Álvaro, Beatriz de-las-Heras, Esther Gil-Alegre, Marta Vicario-de-la-Torre, Rocío Herrero-Vanrell, Irene Teresa Molina-Martínez

**Affiliations:** 1 Department of Pharmaceutics and Food Technology, Faculty of Pharmacy, Complutense University of Madrid (UCM); IdISSC, Plaza Ramón y Cajal s/n, Madrid, 28040 Spain; 2 Innovation, Therapy and Pharmaceutical Development in Ophthalmology (InnOftal) Research Group, UCM, Madrid, Spain; 3 Institute of Industrial Pharmacy (IUFI), Faculty of Pharmacy, UCM, Madrid, Spain; 4 Ramón Castroviejo Ophthalmologic Research Institute, Faculty of Medicine, Universidad Complutense de Madrid, Madrid, Spain; 5 Department of Ophthalmology, Hospital Clínico San Carlos, Madrid, Spain; 6 Clínica Rementería, Madrid, Spain; 7 Medicine and Animal Surgery Department, Faculty of Veterinary, UCM, Complutense Veterinary Clinical Hospital, Madrid, Spain; 8 Department of Pharmacology, Pharmacognosy and Botany, Faculty of Pharmacy, Complutense University of Madrid (UCM), Madrid, 28040 Spain

**Keywords:** Liposomes, Cyclosporine A, Sodium hyaluronate, Topical ocular formulation stability, Ocular surface, Dry eye disease

## Abstract

The prevalence of dry eye disease (DED) is rising globally, increasingly affecting young adults. Although artificial tears are commonly used, unfortunately they do not address the inflammatory component of the disease. For this reason, in patients whose symptoms persist despite the use of artificial tears, anti-inflammatory agents such as the immunosuppressant cyclosporine A (CyA) are clinically indicated to reduce the underlying inflammation in DED. In this study, a CyA-loaded liposomal formulations composed of analogous components to the lipid and aqueous layers of the natural tears (CyA-lipo) and dispersed in an aqueous solution of sodium hyaluronate (CyA-lipo-NaHa) has been developed. Formulations were analysed for physicochemical properties, and in vitro tolerance using human corneal and conjunctival cell lines over a short-term stability study at 25 °C and 2–8 °C. In vivo tolerance was assessed in rabbits, and therapeutic efficacy was evaluated in dogs diagnosed with DED. Average vesicles’ size resulted in 204.2 ± 4.3 nm and 198.7 ± 6.0 nm for CyA-lipo and CyA-lipo-NaHa respectively. Both formulations rendered osmolarity values close to 200 mOsm and surface tension values below 31 mN/m. The addition of sodium hyaluronate produced an increase of viscosity with values of 6.030 ± 0.316 mPa·s for CyA-lipo-NaHa and 0.950 ± 0.073mPa·s for CyA-lipo. Cell viability was above 80% in corneal and conjunctival cells, and no signs of ocular surface damage in rabbit were observed. In dogs, ocular surface parameters, Schirmer’s test values in particular, improved significantly after two months of treatment (< 13 mm/min before and higher than15 mm/min in all animals after treatment). This liposomal formulation demonstrates optimal physicochemical properties, biocompatibility, and therapeutic efficacy, supporting its potential as an optimised treatment for DED.

**Graphical Abstract:**

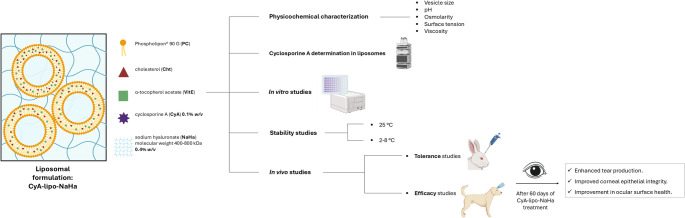

## Introduction

Dry eye disease (DED) is one of the most prevalent ocular conditions globally, affecting millions of people. The Tear Film & Ocular Surface Society Dry Eye Workshop II (TFOS DEWS II) Epidemiology Report, published in 2017 and synthesizing international studies available up to that time, reported wide variation in adult DED prevalence across regions and case definitions (5% − 30%) with prevalence increasing with age and being higher in women [1]. In Spain, the PrevEOS population-based survey conducted in 2022 estimated a DED prevalence of 16.6% using Women’s Health Study (WHS) symptom-based criteria and 22.5% using Beijing Eye Study (BES) criteria. The study reported higher prevalence among women and older adults, but also a notably high prevalence of DED symptoms among young adults aged 18 to 29, despite low rates of clinical diagnosis. PrevEOS highlighted, consistent with TFOS DEWS II findings, the urgent need for improved awareness, diagnosis, and management of DED across the general population [2]. DED is a multifactorial disease characterised by a loss of homeostasis of the tear film, leading to inadequate lubrication of the ocular surface. Common symptoms include dryness, irritation, redness, a sensation of a foreign body, and, in severe cases, pain and blurred vision [3]. The pathogenesis of this disease involves tear film instability, hypertonicity, and a significant inflammatory component. These factors lead to alterations in conjunctival goblet cells, lacrimal glands, and Meibomian glands, ultimately affecting the concentration of electrolytes, mucin, and lipids in the tear film [4]. The persistence of the inflammatory response, in particular, establishes a self-perpetuating vicious cycle that worsens chronic symptoms and hinders both the healing process and the restoration of homeostasis [5, 6]. The therapeutic approach is generally tailored based on the underlying cause, disease severity, and the patient’s response to treatment. [7]. Currently, no definitive treatment exists, and available interventions are aimed exclusively at alleviating symptoms [8].

According to the TFOS DEWS II, artificial tears represent the first intervention for the management of DED [9]. These formulations are designed to replicate the lubricating and protective functions of natural tears, offering symptomatic relief and safeguarding the ocular surface. Artificial tear products range from basic aqueous solutions to more advanced formulations including aqueous and lipid components, the latter tailored to stabilise the lipid layer of the tear film [10–12]. Also, significant proportion of commercial tear preparations contains polymeric and viscosity-enhancing components that help extend the retention time of the formulation on the ocular surface while also providing lubrication. As a matter of fact, the inclusion of polymers stabilises the aqueous-mucin layer of the tear film, thereby reducing surface water evaporation [13]. Furthermore, hyaluronic acid, in the form of sodium hyaluronate (NaHa), has been shown to play a role in the inflammatory response, exhibit antioxidant and healing properties, and promote epithelial cell proliferation in the cornea and conjunctiva [14].

When the DED symptoms fail to improve with artificial tears, it becomes necessary to consider alternative treatments, such as autologous serum and, subsequently, anti-inflammatory agents. In fact, the immune system plays a crucial role in DED, and numerous studies have demonstrated the efficacy of topical glucocorticoids in attenuating the associated inflammatory mechanisms [15]. Topical prednisolone, dexamethasone, fluorometholone, and medroxyprogesterone are the most used [16–18]. While these corticosteroids effectively reduce inflammation, their use is typically reserved for short term treatments or severe cases of DED, due to the risk of serious adverse effects. Consequently, non-steroidal immunomodulators have emerged as an alternative or adjunct treatment strategy, targeting the immunological aspects of the disease. Among these, cyclosporine A (CyA) is the most well-known, having been widely used for moderate to severe cases since its FDA approval in 2003 [19].

The immunomodulatory activity of CyA is attributed to calcineurin inhibition after binding cyclophilin in T lymphocytes, which prevents dephosphorylation and nuclear translocation of nuclear factor of activated T cells (NFAT), downregulating interleukin-2 (IL-2), interleukin-4 (IL-4) transcription and T-cell activation. On the ocular surface, CyA reduces pro-inflammatory mediators (e.g., IL-1β, tumor necrosis factor-α, IL-6) and adhesion molecules (intercellular and vascular cell adhesion molecules 1), exerts anti-apoptotic effects and increases conjunctival goblet-cell density, collectively restoring tear-film homeostasis. Clinically, improvements in signs and symptoms of DED can begin within approximately 4–6 weeks, with more robust changes generally evident by 3–6 months [20–24]. However, the formulation and delivery of this drug to the ocular tissues are challenging due to its relatively high molecular weight and low water solubility. Indeed, CyA’s strong affinity for the lipid phase over the aqueous environment further contributes to its poor bioavailability. For all these reasons, CyA has frequently been prescribed as suspension in compounded formulations in the past years [25]. Since several years ago, eye drops with lipid components containing CyA at concentrations of 0.05% w/v to 0.1% w/v are available on the market. Among the most prominent are Restasis^®^, an oil-in-water emulsion containing 0.05% w/v CyA, Cequa^®^, a 0.09% w/v CyA nanomicellar formulation, Ikervis^®^ a 0.1% w/v CyA cationic nanoemulsion, particularly indicated in cases of severe keratitis associated with DED [26, 27]. Some individuals have reported side effects such as conjunctival congestion, tingling sensations, itching, redness, stinging, and a burning sensation which can significantly hinder treatment adherence, particularly concerning in chronic conditions like DED [28, 29]. Despite the available treatments, there is a critical need for novel therapies that improve CyA efficacy, uptake and bioavailability, while also minimizing side effects and ensuring good patient tolerability. Consequently, the development of new topical formulations is closely linked to creating a pharmaceutical system that are safe for the ocular surface and enhance the quality of the precorneal tear film in patients with DED. Given that the lipid layer of the precorneal tear film is primarily composed of phospholipids, formulations incorporating these components represent a significant advancement. In this regard, liposomes present a promising solution to effectively meet these requirements. They can be prepared with similar components to those present in the tear film. Hydrophobic molecules, such as CyA, are incorporated into the liposomal lipid bilayer, while hydrophilic ones, as the case of NaHa, are included within the aqueous core or dispersed in the external medium [30].

Thus, the idea behind this work was to develop an ophthalmic formulation of CyA-loaded liposomes, both with and without the mucoadhesive polymer NaHa, to alleviate DED symptoms, protect and replenish the preocular tear film and promoting an improvement in ocular surface health. To achieve this, liposomal formulations were first characterised and assessed for stability, followed by evaluations of in vitro and in vivo tolerance using corneal and conjunctival epithelial cells and albino rabbits, respectively. Furthermore, in vivo efficacy studies were conducted in dogs diagnosed with moderate to severe DED to investigate potential improvements in ocular surface health.

## Materials and methods

### Chemicals

Phospholipon^Ⓡ^*90 G (PC) (CAS 97281-47-5*, ≥ 94%) was provided by Lipoid GmbH (Ludwigshafen, Germany). Cyclosporine A (CyA) (CAS 59865-13-3,* ≥ 97%) was supplied by Fagron Iberica S.A.U. (Barcelona*, Spain). Cholesterol (Cht) (CAS 57-88-5, ≥ 99%), α-*tocopherol acetate (VitE) (CAS 7695-91-2*, ≥ 96%), boric acid (CAS 10043-35-3, ≥ 99.5%) sodium tetraborate decahydrate (CAS 1303-96-4, ≥ 99.5%),* were purchased from Sigma-Aldrich (Madrid*, Spain). D-(+)-trehalose dihydrate (CAS 6138-23-4) was obtained from Cymit Química (Barcelona, Spain). Sodium hyaluronate ophthalmic grade (NaHa) (molecular weight 400–800 kDa) was supplied by Abaran Materias Primas S.L. (Villaviciosa de Odon, Spain). All solvents used,* including chloroform*, were of HPLC grade (Panreac Química S.A., Madrid, Spain). Milli-Q^*®*^ purified water was used, *obtained from a Milli-Q*^®^* Gradient A10 system.*

### Liposomes elaboration

CyA-loaded liposomes were prepared according to a previously established protocol [31, 32]. Briefly, a chloroform solution containing PC, Cht, VitE, CyA (8:1:0.08 mg/mL) and 0.2% w/v CyA was vacuum-dried (30 min at 200 mbar, followed by additional 30 min at 100 mbar) to a thin film in an evaporation flask using a rotary evaporator (Büchi^®^, Massó-Analítica, Barcelona, Spain) maintaining the water bath at 33 °C. A dried lipid film was hydrated using a 1.6% w/v trehalose-borate buffered solution, yielding a crude dispersion of liposomes. The liposomal dispersion was then subjected to ultrasonication (Ultrasons-H^®^, J.P. Selecta, Barcelona, Spain) at 5–10 °C to reduce particle size. Following sonication, the dispersion was further homogenized using a high-pressure extruder (Lipex Biomembrane™, Vancouver, BC, Canada) equipped with polycarbonate membrane filters. The extrusion process involved 10 cycles through a 0.8 μm pore size filter, followed by 10 cycles through a 0.2 μm pore size filter, to obtain a sterilized suspension of liposomes with uniform diameters. Two final formulations at 0.1% w/v CyA were prepared by diluting the liposome dispersion with either (i) the trehalose-borate buffered solution to obtain CyA-lipo, or (ii) a solution containing sodium hyaluronate (NaHa) at 0.4% to produce CyA-lipo-NaHa. Blank liposomes were prepared using the same procedure, without the addition of CyA.

### Liposomes’ physicochemical characterization

#### Vesicle size

The vesicle size and polydispersity index (PDI) of the liposomal formulations were characterized using a photon correlation spectroscopy instrument (Zetatrac™, Microtrac^®^, Florida, USA). Measurements were performed in triplicate on samples maintained at 25 °C, following a 30-second vortexing step. Prior to analysis, the samples were diluted in a 1:10 (v/v) ratio.

#### pH

The pH of the samples was measured using a pH meter (Mettler Toledo Svengo, Barcelona, Spain) calibrated with standards at pH equal to 7.00 and 9.00. All measurements were conducted in triplicate at room temperature.

#### Osmolarity

Osmolarity analysis was conducted using a vapour pressure osmometer (K-7000, Knauer GmbH, Berlin, Germany). Measurements were performed at 33 °C and carried out in triplicate.

#### Surface tension

The surface tension of the liposomal formulations was measured in triplicate using the Wilhelmy plate method with a K-11 digital tensiometer (Kruss GmbH, Hamburg, Germany), calibrated beforehand using water (70.0 ± 2.0 mN/m) at 33 °C. Prior to each measurement, the liposomal formulations were also pre-heated to 33 °C and allowed to equilibrate for 3 min.

#### Viscosity

The viscosity of the formulations was evaluated using a RheoStress RS-1 rheometer (RheoStress RS-1, Düsseldorf, Germany) equipped with a 60 mm parallel plate system and a 0.5 mm gap. The rotational speed was gradually increased from 0 to 1000 s⁻¹ in 20 incremental steps. All measurements were conducted in triplicate at 33 °C ± 0.2 °C.

### Cyclosporine A determination in liposomes

The CyA content in liposomal formulations was determined by High-Performance Liquid Chromatography coupled by Ultraviolet detection (HPLC-UV) using a previous analytical procedure with some modifications [33]. Following ICH Q2(R2) and Food and Drug Administration (FDA) guideline (“Validation of chromatographic methods” [34, 35], linearity was evaluated by preparing six calibration levels at 50, 100, 150, 200, 250 and 300 µg/mL. Each level was injected once per sequence on three independent days (*n* = 18 total observations). Calibration curves were generated by plotting peak area versus anal yte concentration. Linearity was assessed by ordinary least-squares regression, reporting the slope, intercept, and correlation coefficient; *R* >0.999 was required to confirm linearity. To demonstrate accuracy and precision (repeatability and intermediate precision) of the HPLC assay for Cyclosporine A within the intended working range, two concentration levels: 100 and 250 µg/mL were evaluated. For each level, six independent samples per day were prepared and analysed on three separate days (*n* = 18 per level). Results are expressed as percent recovery relative to nominal concentration with an acceptance limit of ≤ 3.0%. The chromatographic analysis was performed using a Tracer Extrasil^®^ ODS column (5 μm, 25 × 0.4 cm; Technokroma S.A., Barcelona, Spain) as the stationary phase, maintained at 40 °C throughout the analysis. The isocratic mobile phase consisted of acetonitrile and water (95:5, v/v), pumped at a flow rate of 1 mL/min. CyA detection was carried out at 210 nm. Samples were stored refrigerated in the carousel. CyA retention time was detected approximately at 4 min. Data acquisition and processing were conducted using Empower™ chromatography software (Waters, Barcelona, Spain). For CyA analysis, liposome samples were dissolved by dilution in acetonitrile, followed by vortexing for 3 min to ensure complete dissolution. To confirm the absence of precipitates, the samples were centrifuged at 15,000 rpm for 15 min at 10 °C. The organic supernatant was then carefully collected for analysis.

### Chloroform determination as residual solvent in liposomes

Residual chloroform in blank (non-loaded CyA) liposomal formulations was quantified using Headspace Solid Phase Microextraction coupled with Gas Chromatography-Mass Spectrometry (HS-SPME-GC-MS). The analyses were conducted on an Agilent 7890B gas chromatograph paired with a 5977B single quadrupole mass spectrometer, equipped with a VF-624ms column (1.4 μm, 60 cm × 250 μm; Varian Inc., Madrid, Spain). Calibration standards (0.5–10 ppm) and samples (100–3000 µL) were placed in 20 mL headspace crimp-sealed vials (Agilent, Santa Clara, CA, USA), and the total volume was adjusted to 6 mL with ultrapure water. Headspace equilibration was achieved by incubating the vials at 280 °C for 30 min. After equilibration, 1 mL of the gas phase was injected in pulsed split mode (10:1) into a helium carrier gas flow of 1 mL/min. The temperature program for the gas chromatograph began at 35 °C (held for 15 min), followed by a temperature ramp of 2 °C/min to 50 °C, and then a ramp of 20 °C/min to 240 °C, with a total run time of 43 min. Mass spectrometric detection was performed using electron impact ionization (70 eV), scanning over a mass range of 25 to 650 Da. Quantification was achieved using calibration curves, which were freshly prepared and reinjected at the start of each analysis. The residual chloroform concentration in the formulations was determined using samples equivalent to 10 mg of material, diluted to 6 mL with ultrapure water. The limit of quantification (LOQ) for the method was approximately 0.5 ppm.

### In vitro studies

#### Cell lines and culture conditions

Two cell lines were utilized in this study: human corneal limbal epithelial cells (HCLE), kindly provided by Dr Ilene K. Gipson (Schepens Eye Research Institute, Harvard Medical School, Boston, MA, USA), and human conjunctival epithelial cells (IOBA-NHC) obtained from the Applied Ophthalmology Institute (IOBA, Valladolid, Spain). HCLE cells were cultured in keratinocyte serum-free medium supplemented with a human corneal growth supplement, which includes the necessary growth factors, hormones, and tissue extracts (Life Technologies^®^, Madrid, Spain). IOBA-NHC cells were maintained in a DMEM/F-12 basal growth medium supplemented with 2% penicillin-streptomycin mixture, inactivated foetal bovine serum, bovine pancreas insulin, hydrocortisone, fungizone (Gibco^®^, Life Technologies, Barcelona, Spain), and cholera toxin (Gentaur^®^, Kampenhout, Belgium). Both cell lines were incubated in 75 mL flasks at 37 °C in a humidified atmosphere with 5% CO₂.

#### Cell viability studies

HCLE and IOBA-NHC were seeded into 96-well plates at a density of 10,000 cells per well and cultured overnight (16 h) at 37 °C in an incubator. The following day, cells were treated with liposomal formulations for different incubation periods: 15 min, to simulate short-term ocular exposure (considering the estimated ocular residence time after instillation is approximately 5 min), 1 h and 4 h, to represent prolonged exposure conditions [32, 36]. Afterwards, the culture medium was removed, and fresh growth medium containing the water-soluble tetrazolium salt 3-(4,5-dimethylthiazol-2-yl)-2,5-diphenyltetrazolium bromide (MTT) (Sigma-Aldrich, Madrid, Spain) was added to each well (200 µL/well, 0.5 mg/mL). Cells were then incubated for an additional 3 h at 37 °C. The medium was subsequently removed by careful aspiration, and dimethyl sulfoxide (DMSO, Sigma-Aldrich, Madrid, Spain) (100 µL/well), was added to solubilize the resulting water-insoluble formazan. Cell viability was assessed by measuring absorbance at 550 nm using a microplate reader (model 6010152EU, Digiscan, Eugendorf, Austria). Viability was expressed as a percentage relative to the negative control (cells incubated with supplemented basal growth medium), which was set at 100%. Benzalkonium chloride (BAK, Sigma-Aldrich, Madrid, Spain) (0.005% w/v), a common ocular preservative known for its cytotoxic effects on the ocular surface, was used as a positive control to induce cell death [37, 38].

### Stability studies

Stability studies were conducted on three independent batches under two storage conditions: room temperature (RT) (25 °C, which corresponds to the standard room temperature defined for climatic zone II) and 2–8 °C, over a total period of 6 months. At predefined time points, the physicochemical properties and in vitro tolerance of the liposomal formulations were assessed, along with any potential degradation of the active ingredient (Table 1).

#### Liposomes’ physicochemical characterization under different storage conditions

The physicochemical parameters described in the “Liposomes’ physicochemical characterization” section were evaluated using the same protocols for both CyA-lipo and CyA-lipo-NaHa at three prearranged time points: 0 (freshly prepared), 1 month, and 6 months.

#### Determination of kinetic parameters and any potential degradation of cyclosporine A included in liposomes

To assess the impact of storage temperature on CyA content, the active ingredient in CyA-lipo was quantified and analysed using HPLC-UV, following the method described in the “Cyclosporine A determination in liposomes” section. Measurements were taken at 0 (freshly prepared), 15, 30, 90, and 180 days after storage. Kinetic calculations for CyA degradation in liposomal formulations were performed by plotting the natural logarithm of the remaining drug percentage ($\:ln\left({C}_{t}/{C}_{0}\right)$) versus time (days) under the two storage conditions assayed. Degradation parameters, including the apparent degradation rate constant ($\:k$), half-life ($\:{t}_{1/2}$), which is the time required for 50% degradation, and the time at which 90% of the initial drug concentration remains ($\:{t}_{90}$) were determined. The degradation rate constant ($\:k$) was derived from the slope of the regression (Eq. 1) where$\:{C}_{0}$is the initial concentration and$\:{C}_{t}$is the concentration at time$\:t$.

$$\:ln\left({C}_{t}/{C}_{0}\right)=\:-kt$$

The half-life ($\:{t}_{1/2}$) of CyA in the liposomal formulation was calculated using Eq. 2, where$\:{C}_{50}=\:{C}_{0}/2$.

$$\:{t}_{1/2}=\:\frac{ln\left({C}_{0}/{C}_{50}\right)}{k}$$

Similarly, $\:{t}_{90}$was determined by substituting$\:{C}_{t}=0.9\:\bullet\:\:{C}_{0}$into the Eq. 3.

$$\:{t}_{90}=\:\frac{ln\left({C}_{0}/{C}_{90}\right)}{k}$$

All analyses were conducted in triplicate, and average values were used for the study.

#### In vitro tolerance under different storage conditions

In vitro tolerance was evaluated by assessing the cell viability of each formulation under the two different storage conditions (RT and 2–8 °C) assayed. Measurements were taken for freshly prepared samples and at 1, 3, and 6 months after preparation, across three incubation times, as described in the “Cell viability studies” section. Analyses were performed on both HCLE and IOBA-NHC cell lines.

**Table 1:** List of parameters evaluated for stability studies

Storage conditions	Parameters evaluated	0 days (freshly prepared)	15 days	30 days	90 days	180 days
RT / 2–8 °C	pH	X		X		X
	Osmolarity	X		X		X
	Surface tension	X		X		X
	Viscosity	X		X		X
	Vesicle size	X		X		X
	CyA content *	X	X	X	X	X
	In vitro tolerance	X		X	X	X

* CyA content determined only in CyA-lipo formulation

### In vivo studies

In vivo experiments were conducted in compliance with Spanish regulations on animal experimentation (RD 53/2013 of February 1, 2013, as amended by RD 118/2021 of February 23, 2021), the European Council Directive (86/609/EEC), and the Association for Research in Vision and Ophthalmology (ARVO) Statement on the Use of Animals in Ophthalmic and Vision Research. The procedures observed for tolerance studies were approved by the Animal Experimentation Ethics Committee of the Complutense University of Madrid (PROEX 091.5/21). All institutional and national guidelines for the care and use of laboratory animals were followed. Ocular tolerance studies were carried out at the animal facility of the Faculty of Medicine (Complutense University of Madrid) using male albino New Zealand rabbits weighing 2.0–2.5 kg (Granja San Bernardo, Tulebras, Spain). Rabbits were housed individually in cages, provided with rabbit pellets and water ad libitum, and maintained under a 12-hour light-dark cycle.

Subsequently, efficacy studies were conducted on client-owned dogs diagnosed with immune-mediated DED or DED associated with endocrine disorders. These dogs were examined at the Ophthalmology Service of the Clinical Veterinary Hospital (Complutense University of Madrid). The main inclusion criteria for the study were a Schirmer’s test (STT) result of below 15 mm/min in both eyes and the presence of clinical signs linked to DED [39, 40]. All procedures and protocols were carried out by specialist veterinarians. Informed written consent was obtained from all dog owners prior to enrolment. In both studies, sample size determination adhered to the principles of the 3Rs.

#### Tolerance studies

Tolerance studies were performed as previously described [41–43]. Briefly, the tolerance of the developed liposomal formulations was assessed by applying repeated topical instillations of 30 µL every 30 min during a total of 6 h to the animals’ test eye (n = 6 eyes), the same throughout all the procedure, with a total of 12 applications. As control, the contralateral eye was instilled with isotonic saline solution following the same procedure (n = 6 eyes). Signs of discomfort, secretions, swelling, and changes in both the cornea and conjunctiva after fluorescein staining were macroscopically assessed by a trained ophthalmologist right before the beginning of the assay and at intervals of 24, 48, and 72 h following the initial administration. Ocular surface evaluation was conducted according to a modified protocol previously described in the literature (Table 2) [44].

**Table 2:** Scoring system for the tolerance studies of the liposomal formulations tested

Grade	Discomfort	Cornea	Conjunctiva	Discharge	Iris
0	No reaction	No ulceration or opacity	No alterations	No discharge	Normal
1	Blinking	Scattered or diffuse areas of opacity	Mild hyperaemia/mild oedema	Mild discharge without moistened hair around animal’s eyes	Markedly deepened rugae, congestion, swelling, iris reactive to light
2	Enhanced blinking/intense tearing/vocalizations	Easily discernible translucent area	Moderate hyperaemia/moderate oedema	Intense discharge with moistened hair around animal’s eyes	Haemorrhage, gross destruction, no reaction to light
3		Nacreous area; no details of iris visible; size of pupil barely discernible	Intense hyperaemia/intense oedema/haemorrhage		
4		Opaque cornea; iris not discernible through the opacity			

#### Efficacy studies

A two-month observational, prospective, single-arm clinical study was performed on client-owned dogs under routine veterinary care of the Clinical Veterinary Hospital exhibiting moderate to severe DED symptoms (*n* = 20). Comparisons were within-subject (baseline versus follow-up) on pre-defined clinical outcomes; no blank/vehicle/disease-only groups were implemented due to welfare and practical considerations in this clinical context. Based on Schirmer’s test (STT) values, the animals were divided into two cohorts. Group I (mild cases) included dogs with STT values between 10 and 15 mm/min, whereas Group II (moderate to severe cases) comprised dogs with STT values below 10 mm/min.

For this preliminary evaluation, STT was performed without anaesthesia using sterile strips, which were placed on the palpebral conjunctiva of the lower eyelid for 60 s with the eyes closed. Tear volume, measured in mL of the moistened strip, was recorded [39, 40]. A drop (30 µL) of CyA-lipo-NaHa was topically administered to both eyes 3 times per day for 60 consecutive days by the dogs’ owners in a home setting. A complete ophthalmic examination was assessed at two time points: before the beginning of the efficacy studies (time I) and after 60 days of treatment (time II) in both groups. This examination, performed by veterinarians, included STT, assessment of fluorescein corneal staining, evaluation of ocular discharge presence and severity, measurement of corneal thickness using ultrasound pachymetry (SP100, Tomey, Nürnberg, Germany), and corneal sensitivity assessment with a Cochet-Bonnet esthesiometer (Luneau Ophtalmologie, Paris, France).

### Statistical analysis

Each liposomal formulation was prepared in three different batches, and each batch was analysed in triplicate. Statgraphics Centurion 19© (Statgraphics Technologies, Inc., The Plains, VA, USA) software was used for statistical determinations. Statistical differences between groups were assessed using one-way analysis of variance (ANOVA) for in vitro experiments and Student’s t-test for in vivo data. McNemar’s test was applied to evaluate changes in fluorescein corneal staining and ocular discharge before and after treatment in the dogs under the study. A probability value lower than 0.05 (*p* < 0.05) was considered statistically significant (ns; *: *p* ≤ 0.05; **: *p* ≤ 0.01; ***: *p* ≤ 0.001).

## Results

### Liposomes’ physicochemical characterization

**Fig. 1:**
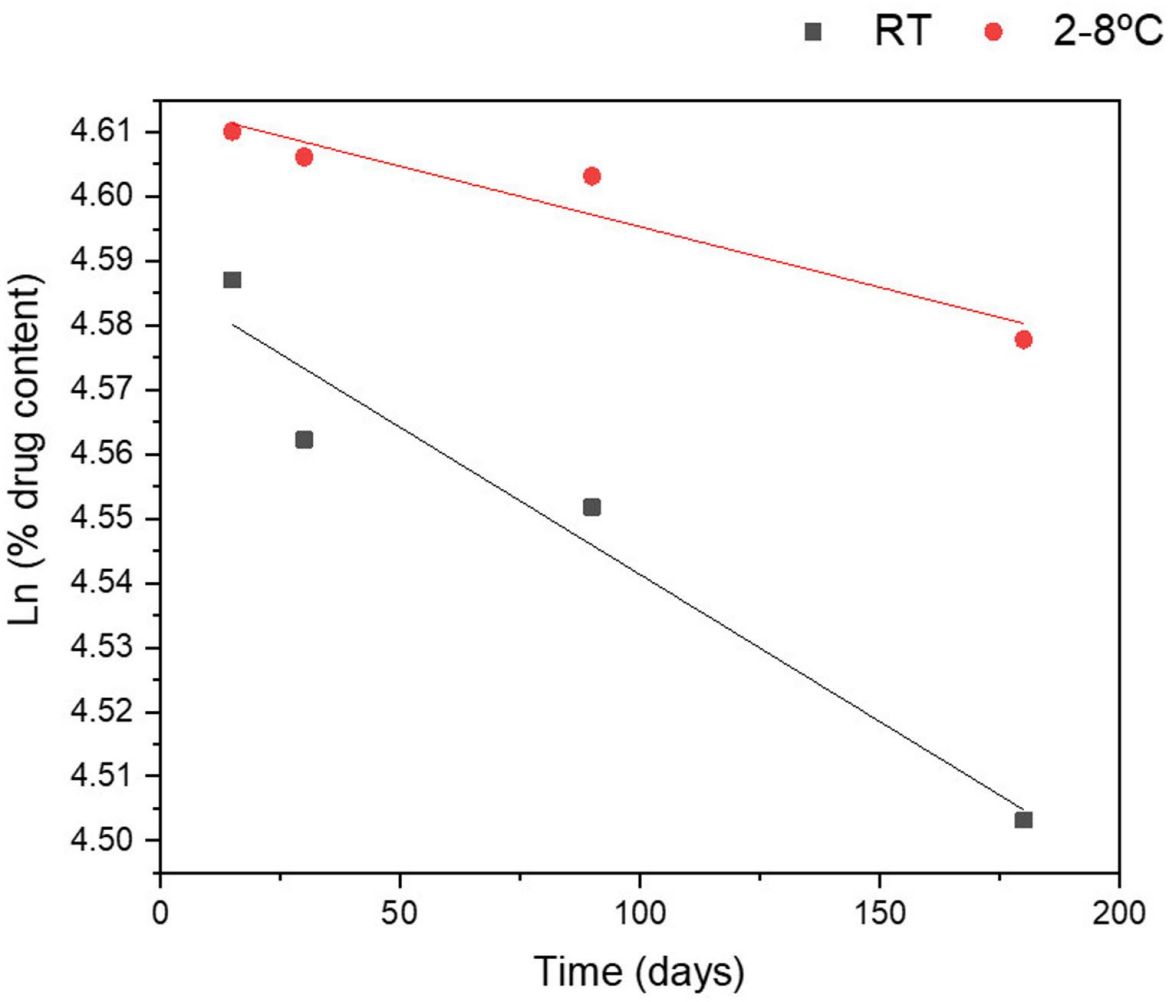
Graphical representation of the natural logarithm of CyA content (%) over time from stability studies conducted with the CyA-lipo formulation

Both CyA-lipo and CyA-lipo-NaHa exhibited a unimodal size distribution (Fig. 1), with average vesicle sizes of 204.2 ± 4.3 nm and 198.7 ± 6.0 nm, respectively. The incorporation of NaHa did not significantly affect vesicle size (p >0.05). Both formulations had similar pH values, consistent with the physiological pH of the ocular surface. Moreover, both formulations are hypotonic, with osmolarity values close to 200 mOsm/L (p >0.05). The surface tension of both formulations resulted lower than that of human tears (43.60 ± 2.70 mN/m) [45, 46]. Rheological analyses showed that CyA-lipo had a comparable viscosity to water and human tears (0.950 ± 0.073mPa·s), whereas the viscosity of CyA-lipo-NaHa was more than six times higher. A summary of these parameters is presented in Table 3.

**Table 3:** Physicochemical properties of the liposomal formulations developed

Formulation tested	Mean vesicle size [nm]	pH	Osmolarity [mOsm/L]	Surface tension [mN/m]	Viscosity [mPa·s]
*CyA-lipo*	*204.2 ± 4.3*	*7.36 ± 0.03*	*203.9 ± 9.3*	*29.8 ± 1.4*	*0.950 ± 0.073*
*CyA-lipo-NaHa*	*198.7 ± 6.0*	*7.36 ± 0.04*	*200.5 ± 2.8*	*28.9 ± 0.8*	*6.249 ± 0.316*

### Cyclosporine A determination in liposomes

The concentration of CyA in CyA-lipo was determined using the procedure described in the “Cyclosporine A determination in liposomes” section. This HPLC method used for CyA quantification was validated in accordance with ICH Q2(R2) and FDA guidance. Linearity across 50–300 µg/mL was confirmed by an intercept not significantly different from zero (t-test, *p* ≥ 0.05) and a highly significant area–concentration regression (ANOVA, *p* < 0.001). Regarding accuracy, neither day nor concentration affected recovery (%) at the 95% confidence level (ANOVA: day *p* = 0.374; concentration *p* = 0.052) at both levels studied (100 and 250 µg/mL). The pooled estimate demonstrated accuracy of 100.2% (95% Confidence Intervals: 99.6–100.8%), meeting the predefined acceptance limit of ± 3% across both levels consistent with ICH Q2(R2). Precision was evaluated by pooling data across the two concentration levels (100 and 250 µg/mL). The coefficients of variation were 1.7% for repeatability (within-day) and 1.7% for intermediate precision (between-day), meeting the predefined acceptance limits (≤ 2.0% within-day; ≤ 3.0% across days).

Applying this method, in the freshly prepared formulation, the CyA content was 96.25 ± 3.18 µg/mL, corresponding to an entrapment efficiency of 96.3 ± 3,3%, based on the theoretical drug-to-lipid ratio used during preparation (50 µg/mg).

### Chloroform determination as residual solvent in liposomes

Three independent batches produced using the protocol described at in the “Liposomes elaboration” section. were analysed. In all cases, the residual chloroform content was below 10 ppm (0.5, 0.9 and ≤ 0.5 ppm) and remained below the established limit (< 60 ppm) for ophthalmic topical formulations in the ICH Q3C (R9) guidelines for residual solvents [47].

### In vitro studies

**Fig. 2:**
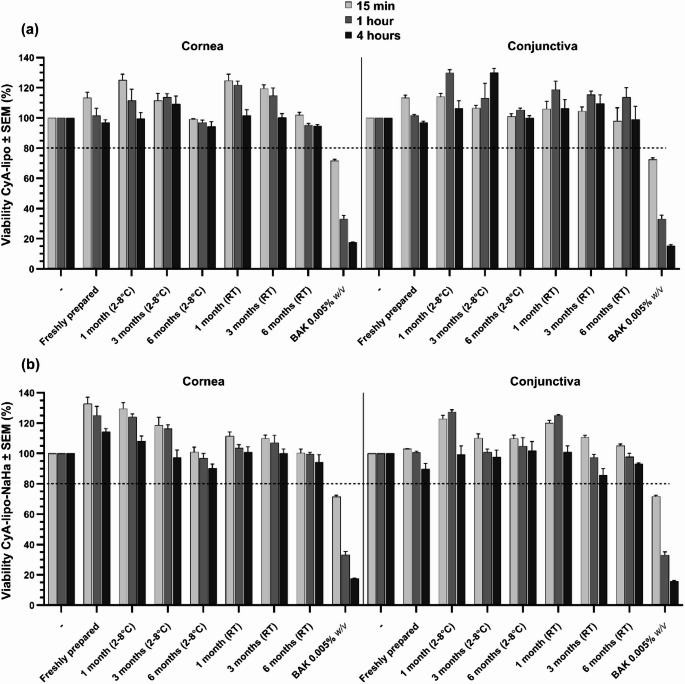
Corneal and conjunctival cell viability (mean expressed as percentage ± SEM) for control solutions, CyA-lipo (**a**), and CyA-lipo-NaHa (**b**) freshly prepared, and stored at RT and 2–8 °C for 6 months

In viability studies performed on the human corneal epithelial cell line (HCLE), both CyA-lipo and CyA-lipo-NaHa exhibited viability values close to 100% across all tested exposure times (15 min, 1 h, and 4 h), indicating excellent cellular tolerance (Fig. 2). Similar results were obtained in the human conjunctival cell line (IOBA-NHC).

### Stability studies

#### Liposomes’ physicochemical characterization under different storage conditions

Both liposomal formulations, CyA-lipo and CyA-lipo-NaHa, demonstrated high stability, with no significant changes in their physicochemical properties after 6 months of storage under the two evaluated conditions (Table 4). Regarding vesicle size, pH, osmolarity, surface tension, and viscosity, the values of both formulations remained constant during the 6-months follow-up under either refrigerated or at RT (*p* > 0.05).

**Table 4:** Physicochemical parameters evaluated in the stability studies for the two liposomal formulations developed, CyA-lipo and CyA-lipo-NaHa

Formulation tested	Storage conditions	Mean vesicle size [nm]	pH	Osmolarity [mOsm/L]	Surface tension [mN/m]	Viscosity [mPa·s]
CyA-lipo	1 month (RT)	200.4 ± 6.0	7.39 ± 0.01	201.1 ± 1.7	29.2 ± 1.3	0.916 ± 0.007
	6 months (RT)	196.4 ± 3.2	7.33 ± 0.09	196.6 ± 2.8	29.8 ± 1.3	0.909 ± 0.061
	1 month (2–8 °C)	199.1 ± 3.2	7.34 ± 0.04	201.8 ± 2.7	29.3 ± 0.8	0.959 ± 0.057
	6 months (2–8 °C)	201.7 ± 0.5	7.38 ± 0.02	199.7 ± 2.7	30.1 ± 1.4	0.886 ± 0.035
CyA-lipo-NaHa	1 month (RT)	204.7 ± 8.3	7.33 ± 0.04	204.6 ± 4.9	29.2 ± 0.4	6.261 ± 0.190
	6 months (RT)	199.9 ± 1.6	7.40 ± 0.02	200.4 ± 6.4	30.2 ± 0.8	6.318 ± 0.079
	1 month (2–8 °C)	200.7 ± 1.2	7.37 ± 0.03	202.6 ± 2.1	28.8 ± 0.9	6.253 ± 0.123
	6 months (2–8 °C)	198.7 ± 96	7.41 ± 0.01	197.2 ± 2.1	29.6 ± 0.7	6.273 ± 0.248

#### Determination of kinetic parameters and any potential degradation of cyclosporine A included in liposomes

Stability studies were carried out on CyA-lipo under dark conditions and either 2–8 °C or RT. Table 5presents the results obtained at the different time points evaluated (15 days, 1, 3, and 6 months). Data were processed using the drug contents, expressed as a percentage of the initial concentration at time zero.

The chemical stability of CyA was assessed under the two storage conditions previously mentioned and quantified using HPLC-UV (“Cyclosporine A determination in liposomes” section). A linear correlation was observed when plotting $\:ln\left({C}_{t}/{C}_{0}\right)$versus time, both at room temperature (r^2^ = 0.925) and under refrigeration (r^2^ = 0.939), indicating that CyA degradation followed first-order kinetics (Fig. 1). The kinetic parameters were calculated using Eqs. 1, 2, and 3, respectively (Table 6). The highest degradation rate occurred at RT, where the $\:k$ value was 2.5 times greater than that calculated under refrigerated conditions.

**Table 5:** CyA concentration (%) (mean ± SD) in CyA-lipo stored under refrigeration and at room temperature throughout the stability studies

Storage conditions	Time points	CyA concentration [%]
2–8 °C	15 days	100.5 ± 6.2
	1 month	100.1 ± 3.1
	3 months	99.8 ± 4.7
	6 months	97.3 ± 2.7
RT	15 days	98.2 ± 10.4
	1 month	95.8 ± 9.7
	3 months	94.8 ± 14.0
	6 months	90.3 ± 2.0

**Table 6:** Kinetic parameters evaluated for CyA-lipo stored under refrigeration and at room temperature for 6 months

Storage conditions	$\:k$ [months^− 1^]	$\:{t}_{1/2}$ [months]	$\:{t}_{90}$ [months]
RT	0.01379	50.6	6.4
2–8 °C	0.00564	122.9	20.3

#### In vitro tolerance under different storage conditions

The results of cell survival after 1-, 3- and 6-months exposures to both CyA-lipo and CyA-lipo-NaHa are presented in Fig. 2for corneal and conjunctival cells. After incubation times tested, 15 min, 1h and 4 h, no cell death was detected for any storage condition (time and temperature) in both cell lines studied. When the storage time was increasing, a slight decrease in cell viability was observed, though cell survival resulted always higher than 80% for any test formulation compared to negative control. No differences were observed in the liposomal formulation with the addition of 0.4% w/v NaHa. Concerning the positive control, a 0.005% w/v BAK solution, a significant decrease in cell survival was obtained at every in vitro tolerance test.

### In vivo studies

#### Tolerance studies

Prior to the administration, macroscopic examination of the rabbits’ eyes confirmed healthy conjunctiva and corneas, with no signs of discharge, eyelid swelling, or discomfort. The ocular surface remained unaltered and without any inflammation at all the time points evaluated. The animals which received either CyA-lipo or CyA-lipo-NaHa consistently showed a Grade 0 according to the criteria in Table 2. Comparative analysis with isotonic saline solution showed no significant differences. Detailed evaluation results are presented in Table 7.

**Table 7:** Grading system and results of the in vivo ocular tolerance evaluation in rabbits for both CyA-lipo and CyA-lipo-NaHa

	Isotonic saline solution			CyA-lipo			CyA-lipo-NaHa		
	24 h	48 h	72 h	24 h	48 h	72 h	24 h	48 h	72 h
Discomfort	0	0	0	0	0	0	0	0	0
Cornea	0	0	0	0	0	0	0	0	0
Conjunctiva	0	0	0	0	0	0	0	0	0
Discharge	0	0	0	0	0	0	0	0	0
Iris	0	0	0	0	0	0	0	0	0
Total score	0	0	0	0	0	0	0	0	0

#### Efficacy studies

**Fig. 3:**
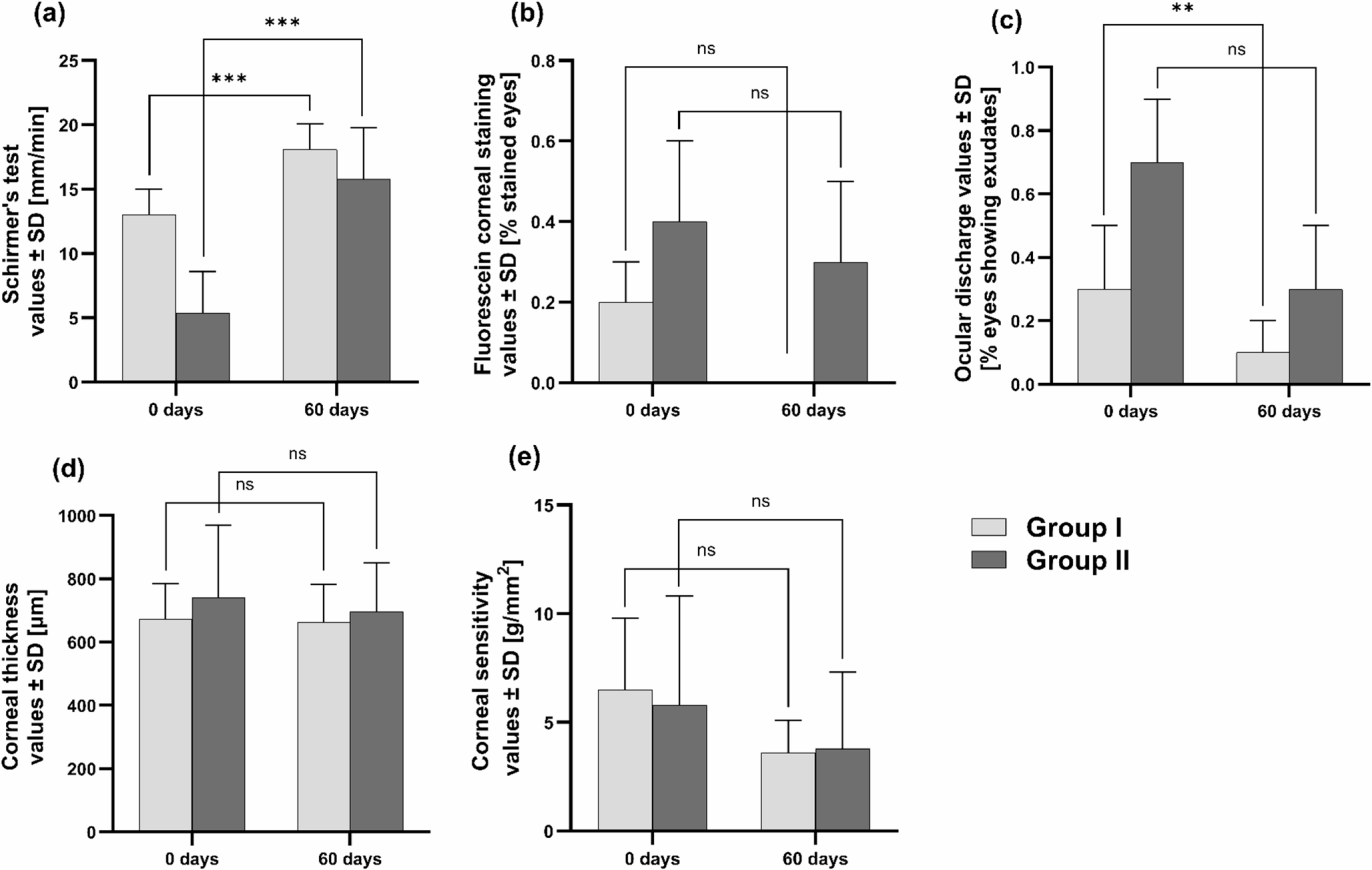
Comparison of STT (**a**), fluorescein corneal staining (**b**), ocular discharge (**c**), corneal thickness (**d**), and corneal sensitivity (**e**) values before and after administration of the evaluated liposomal formulation (CyA-lipo-NaHa) in either Group I or Group II animals (ns; *: *p* ≤ 0.05; **: *p* ≤ 0.01; ***: *p* ≤ 0.001). Note: The bar for Group I in Fig. 3 (b) is not visible because all animals in this group showed no staining (score = 0), indicating complete resolution of the evaluated clinical sign.

Dogs were classified into two cohorts based on disease severity, as determined by STT values, following the criteria described in the “Efficacy studies” section (*n* = 10 for Group I; *n* = 10 for Group II). STT measurements obtained before and after treatment with CyA-lipo-NaHa are presented in Table 8. A statistically significant increase in STT values was observed post-treatment in both groups (*p* < 0.001). After 60 days, mean STT values exceeded 15 mm/min in both cohorts (Fig. 3a). Group I (mild cases) exhibited a 40% increase in STT values, whereas Group II (moderate to severe cases) showed a 192% increase.

Biomicroscopic examination of the ocular surface using a slit lamp and fluorescein staining revealed a general improvement in surface integrity, with all initial and post-treatment positive staining classified as grade 1 (Fig. 3b). In Group I, no positive staining was observed following treatment. However, the differences were not statistically significant in either group (McNemar’s test: *p* = 0.057 for mild cases, *p* = 0.327 for moderate to severe cases). Mucous secretions, typically associated with mucopurulent conjunctivitis, were reduced in both cohorts (Fig. 3c), reaching statistical significance only in Group I (*p* = 0.005; *p* = 0.057 for Group II). Corneal pachymetry data (Fig. 3d) showed a slight reduction in corneal thickness, averaging 2% in mild cases and 4% in moderate to severe cases, though these changes were not statistically significant among the two cohorts (*p* > 0.05). Corneal sensitivity, typically reduced in inflammatory conditions, showed an improving trend post-treatment, but without statistical significance (*p* > 0.05). The pressure required to produce a corneal response decreased by 46% in the mild group and 34% in the moderate to severe group (Fig. 3e). All dog owners expressed satisfaction with the liposome-based treatment and reported a subjective improvement in ocular symptoms, reflecting improved well-being in their dogs.

**Table 8:** In vivo efficacy studies results (mean ± SD) prior and after 60-day treatment with CyA-lipo-NaHa in client-owned dogs exhibiting either mild (*n* = 10) or moderate to severe DED symptoms (*n* = 10)

	Before CyA-lipo-NaHa treatment (0 days)		After CyA-lipo-NaHa treatment (60 days)	
	Group I	Group II	Group I	Group II
Schirmer’s test [mm/min]	13.0 ± 2.0	5.4 ± 3.2	18.1 ± 2.0	15.8 ± 4.0
Fluorescein corneal staining [% stained eyes]	0.2 ± 0.1	0.4 ± 0.2	0.0 ± 0.0	0.3 ± 0.2
Ocular discharge [% eyes showing exudates]	0.3 ± 0.2	0.7 ± 0.2	0.1 ± 0.1	0.3 ± 0.2
Corneal thickness [µm]	673 ± 112	742 ± 228	662 ± 121	696 ± 155
Corneal sensitivity [g/mm^2^]	6.5 ± 3.3	5.8 ± 5.0	3.6 ± 1.5	3.8 ± 3.5

## Discussion

DED remains as a prevalent and challenging condition, largely due to the lack of definitive treatments. As highlighted in the TFOS DEWS II report, there is a pressing need for more effective therapies [9]. Recent data from the PrevEOS study reveal an increasing prevalence of DED in young adults, linked to digital device use and environmental stressors, underscoring the need for early diagnosis and intervention [2]. While artificial tears are often insufficient, the growing use of medicated eye drops, particularly anti-inflammatory agents like CyA, reflects the shift toward more targeted treatments [48]. CyA, despite its established role in DED management, is limited by poor bioavailability, potential side effects and low patient compliance, highlighting the need for novel pharmaceutical formulations that can enhance delivery and improve ocular surface health [49]. In this study, liposomes with similar components of the lipids present in tear film (PC and Cht) were used as a technological platform to improve CyA bioavailability aiming to replenish and protect the ocular surface. In addition to evaluating CyA bioavailability in liposomes, this study also aimed to promote ocular surface restoration through a biomimetic lipid composition [50–54]. Moreover, all major diagnostic parameters for DED were assessed over a long-term follow-up, an aspect not fully addressed in previous studies.

In this article, preliminary experiments were conducted to determine the maximum amount of CyA that could be incorporated into the lipid phase of the liposomes. It was found that CyA concentrations exceeding 5% w/v relative to PC inhibited complete encapsulation, resulting in the formation of crystalline precipitates (data not shown). During formulation development, CyA was incorporated with the other lipophilic components and was entrapped into the liposomal bilayer, as shown in the “Cyclosporine A determination in liposomes” section, in a manner consistent with previous reports [50, 52]. Additionally, the amount of residual chloroform in the liposomal formulations complies with the ICH Q3C (R9) guideline, indicating that the levels pose no safety concerns for ocular administration (< 60 ppm) and that the preparation method is suitable for scale-up for clinical use [47].

PC was the main excipient in the formulation due to its role as the principal component of the lipid layer in the preocular tear film, accounting for approximately 60% of the phospholipids present in Meibomian Glands secretions. From a technological perspective, PC demonstrates superior surfactant properties that contribute significantly to the stabilisation of the formulation and also enables the formation of liposomes that are particularly advantageous due to their established ability to thicken the tear film lipid layer, bind tear lipocalins, and stabilise the ocular surface environment [30, 55]. To further enhance formulation stability, Cht and VitE were incorporated during liposome manufacturing to protect PC from oxidative degradation and improve liposome stability from oxidative degradation [56]. Additionally, a borate buffer was employed in the aqueous phase for both adjusting the pH to physiological levels (~ 7.4, as described in the “Liposomes’ physicochemical characterization” section) and providing antimicrobial protection [57]. Trehalose was incorporated into the borate buffer both to adjust tonicity and to provide osmoprotective effects at the ocular surface. Through water-replacement mechanisms, trehalose mitigates desiccation-induced epithelial damage and inflammatory signaling; these effects have been reported in experimental models and clinical studies of dry eye [58, 59].

Two liposomal formulations were developed and evaluated: one containing 0.4% w/v sodium hyaluronate (NaHa) in the external medium (CyA-lipo-NaHa) and one without NaHa (CyA-lipo). The inclusion of the bioadhesive polymer NaHa was intended to increase formulation viscosity, thereby enhancing ocular residence time and supporting tissue repair [60]. Rheological measurements confirmed this consideration, with CyA-lipo-NaHa exhibiting a viscosity more than six times higher (6.03 ± 0.316 mPa·s) than that of CyA-lipo (0.950 ± 0.073mPa·s). This viscosity range helps prevent visual disturbance and allows the formulation to spread evenly across the tear film [30, 61].

Regarding the other physicochemical parameters evaluated, achieving a vesicle size of approximately 200 nm, as obtained in the current study for both CyA-lipo and CyA-lipo-NaHa, is advantageous. Liposomes within this size range can efficiently penetrate the layers of the corneal epithelium, thus reducing immunogenic reactions, minimising uptake by the mononuclear phagocyte system, and preventing blurred vision following topical application [62, 63]. The surface tension values obtained were lower (~ 30 mN/m) to those typically found in human tears (43.60 ± 2.70 mN/m), suggesting that the liposomal formulations developed can effectively spread across the ocular surface [45, 46]. Additionally, both CyA-lipo and CyA-lipo-NaHa were formulated as slightly hypo-osmolar preparations (~ 200 mOsm/L) to help restore the physiological isotonicity of the tear film in patients with DED, which is characterized by tear hyperosmolarity. In fact, elevated osmolarity in DED is known to upregulate matrix metalloproteinases and pro-inflammatory cytokines, thereby promoting inflammation and inducing apoptosis in corneal and conjunctival epithelial cells [64–66]. In this context, it is important to highlight that NaHa, with the molecular weight used in this research, has previously demonstrated osmoprotective behaviour in an in vitro hyperosmolarity model, reinforcing its beneficial application in DED therapy [67].

Since corneal and conjunctival cells are the most affected in DED, the tolerance of the developed formulations was previously assessed using conjunctival and corneal epithelial cells under short (15 min and 1 h) and long (4 h) exposure conditions, as previously established and validated by our research group [68]. The results showed that, the liposomal formulations developed in this study, maintained cell viability above 80%, indicating good in vitro tolerance. The inclusion of NaHa in the ophthalmic formulations was associated with a slight increase in cell viability, that it is consistent with previous findings [69]. In the present work we did not include blank liposome groups, as their tolerance had already been addressed [62]. The short-term stability study of the liposomal formulations showed no significant changes in the physicochemical properties or cell viability of either formulation, with viability consistently exceeding 80% at both 2–8 °C and RT for 6 months. Moreover, liposomal vesicles managed to protect CyA from degradation (≥ 90% CyA concentration) during the six months evaluated, as formerly proved [70]. Our stability assessment quantified CyA content under defined conditions (≥ 90% at six months) using a validated assay, but it was not stability-indicating and did not resolve potential degradants; likewise, we did not directly map liposome penetration or membrane fusion in ocular tissue. As next steps, an ICH-aligned stability-indicating method, together with ex vivo confocal imaging of cryosectioned eyes using labeled liposomes. Following 12 repeated instillations in rabbits and evaluation at 72 h post-exposure, rabbits showed grade 0 scores for al assessed parameters in CyA-lipo and CyA-lipo-NaHa, without differences versus isotonic saline solution (Table 7), indicating absence of detectable irritation under the study conditions. The formulations resulted well tolerated and considered suitable for topical ophthalmic use, thereby reinforcing the in vitro tolerance findings.

For the efficacy studies, the most comprehensive formulation, CyA-lipo-NaHa was selected, to perform evaluations in dogs with naturally suffering DED. This approach circumvents several limitations associated with experimental animal models, which often fail to replicate the full spectrum of the disease, instead targeting isolated aspects, and do not capture its chronic and multifactorial nature. Spontaneous DED in dogs is therefore regarded as a highly relevant model for evaluating new pharmaceutical formulations under conditions that closely mirror clinical reality [71, 72]. The in vivo efficacy results for CyA-lipo-NaHa confirm its therapeutic potential after two months of treatment, with significant clinical improvements observed across both the cohorts, particularly in cases of moderate to severe one. This finding is particularly noteworthy given that CyA is not a fast-acting drug and typically requires prolonged administration, with standard therapies often extending to six months or longer [73]. We acknowledge the absence of parallel control groups as a limitation of this study. Although, owners reported enhanced comfort and well-being in their dogs, alongside objective clinical improvements, underscore the formulation’s potential to improve quality of life in veterinary patients with chronic ocular disease conditions in veterinary patients. Another limitation of the present study is the absence of a marketed CyA formulation as a direct comparator due to the clinical nature of the study, which therapeutic strategy was to prioritize the use of the most promising formulation, the polymer-enriched liposomal CyA, based on its in vitro performance and expected tolerability, to ensure the best possible care for the animals. Several marketed CyA formulations are available for DED treatment, including Ikervis^®^ (0.1% CyA, Europe), Restasis^®^ (0.05% CyA, USA), and Verkazia^®^ (0.1% CyA). While all have shown clinical efficacy, they are commonly associated with ocular irritation, burning, and patient discomfort, largely attributed to their vehicles (oil in water emulsion and specific oily components for Ikervis^®^ and Verkazia^®^, respectively) and the hydrophobic nature of CyA, which limits bioavailability and requires long-term, frequent application [74, 75]. These challenges are particularly relevant in both human and veterinary patients requiring long-term treatment for dry eye disease. In contrast, the liposomal formulation developed in this study offers an aqueous, non-irritating vehicle, capable of encapsulating CyA while enhancing ocular residence time. The inclusion of the HaNa as a mucoadhesive polymer further improves tear film stability and bioadhesion. These features are particularly relevant in veterinary patients, where treatment adherence and ocular tolerance are critical, and suggest that liposomal CyA may offer a viable alternative to current commercial products.

These findings support the translatability of the liposomal formulations developed as potential DED treatment since they could be capable of maintaining the integrity of the ocular surface while therapeutic efficiency is achieved.

## Conclusions

The liposomal formulations loaded with cyclosporine A developed in this study showed suitable physicochemical characteristics for their application on the eye and optimal in vitro tolerance that remained excellent during a six-month storage. The developed formulations showed good in vivo tolerance and the one with the addition of sodium hyaluronate revealed to be therapeutically efficient in the treatment of symptoms of moderate to severe dry eye disease in dogs.

These liposomal formulations were specifically designed to both mimic and replenish the natural composition of the precorneal tear film, incorporating components that support its structural and functional restoration, such as phospholipids, cholesterol, α-tocopherol acetate and sodium hyaluronate. In addition to this, liposomes act as nanocarriers for cyclosporine A while providing optimal biocompatibility properties. This dual mechanism, structural and pharmacological, could contribute to re-establishing tear film homeostasis and improving the overall condition of the ocular surface and support the translational potential of the formulation as a therapeutic strategy for dry eye disease.

## Data Availability

Datasets and graphs are available from the corresponding authors upon request.
